# Parents’ experiences of participating in an intervention on tobacco prevention in Child Health Care

**DOI:** 10.1186/1471-2431-14-69

**Published:** 2014-03-11

**Authors:** AnnaKarin Johansson, Noomi Carlsson, Helena Almfors, Monica Rosèn, Siw Alehagen

**Affiliations:** 1 Department of Medicine and Health, Division of Nursing Science, Faculty of Health Sciences, Linköping University, Linköping SE58183, Sweden; 2 Department of Clinical and Experimental Medicine, Division of Paediatrics, Linköping University, Linköping, Sweden; 3 Department of Public Health and Medical Care, Jönköping County Council, SE-551 11, Box 1024 Jönköping, Sweden

## Abstract

**Background:**

Child health care is an important arena for tobacco prevention in Sweden. The aim of this study was to describe parents’ experiences from participating in a nursebased tobacco prevention intervention.

**Methods:**

Eleven parents were interviewed using semi-structured interviews. The material was analysed in a qualitative content analysis process.

**Results:**

The analysis emerged four categories; Receiving support, Respectful treatment, Influence on smoking habits and Receiving information. The parents described how the CHC nurses treated them with support and respect. They described the importance of being treated with respect for their autonomy in their decisions about smoking. They also claimed that they had received little or no information about health consequences for children exposed to environmental tobacco smoke (ETS). The findings also indicate that both the questionnaire used and the urine-cotinine test had influenced parents’ smoking.

**Conclusion:**

The clinical implication is that CHC is an important arena for preventive work aiming to minimize children’s tobacco smoke exposure. CHC nurses can play an important role in tobacco prevention but should be more explicit in their communication with parents about tobacco issues. The SiCET was referred to as an eye-opener and can be useful in the MI dialogues nurses perform in order to support parents in their efforts to protect their children from ETS.

## Background

Children who are exposed to environmental tobacco smoke (ETS) have an increased incidence of health problems, such as respiratory symptoms like wheeze and asthma [1,2], middle ear infection and sudden infant death syndrome [2]. Studies have shown that the most common place where children are exposed to ETS is in their homes [3-5]. In Sweden, 14% of eight-month-old children born in 2009 had at least one smoker in the family [6].

Preventing exposure to tobacco smoke in childhood is important. Several interventions have been implemented and evaluated worldwide. There are insufficient data to allow one kind of intervention to be recommended over another [7]. The most effective way for parents to protect children from ETS exposure, apart from quitting smoking, is to smoke outdoors with the door closed [3].

The Child Health Care (CHC) nurse can play an important role in encouraging parents to give up smoking or helping parents to protect their child from ETS. In Sweden, almost 100% of all parents have regular contact with CHC from the child´s birth to the age of six year. The CHC service is free of charge and more than 99% of the Swedish families participate. The CHC nurse is a specialist nurse running the CHC independently, with a paediatrician or general practitioner as consultant. The task of CHC is to support and give advice on issues such as parenthood, breastfeeding, weaning, eating habits, accident prevention and to carry out health check-ups. The health promotion includes tobacco prevention [8]. Previous research indicates that CHC nurses had a positive attitude to tobacco prevention [9]. However, results indicated that parents were not satisfied with tobacco prevention in CHC and expressed a wish to have a dialogue with the CHC nurse about how they could protect their child from ETS exposure [10].

A nurse based intervention was implemented in order to improve tobacco prevention in Child Health Care. One part was to increase parents` awareness of the importance of protecting children from tobacco smoke and support them in their efforts to do so. CHC nurses (n = 22) were recruited and they involved 86 families in which at least one parent smoked. The intention was to reach non-Swedish speaking parents. Therefore, all written material was translated into the 9 languages spoken in the area. The parents participated in this intervention for 12 months. The CHC nurses focused on repeated dialogues with the parents about smoking, its consequences, and how to protect the child from ETS exposure. The nurses were educated in Motivational Interviewing (MI) [11] and used this in their dialogues with parents. They were also supported by an instrument, SiCET (Smoking in Children’s Environment Test), in the dialogues [12,13]. To evaluate the intervention, two urine-cotinine tests from the child, one at inclusion and one after twelve months, were obtained. Cotinine is a metabolite of nicotine and the best objective measure of ETS exposure [14]. The results of the pilot study indicated decreased ETS exposure for children whose parents participated in the intervention and that SiCET worked well as a support in the dialogues [15] The study describing nurses experiences from the intervention showed that SiCET led to increased understanding of the child’s ETS exposure, which could facilitate parents’ behavioural changes [13]. The next crucial step was to study the parents’ experiences from taking part in the intervention.

The aim of this study was to describe parents’ experiences from participating in a nurse based tobacco prevention intervention.

## Method

### Design

A qualitative inductive design with interviews was chosen.

#### *Setting*

The pilot study took place in 17 CHC centers in a county in the south east of Sweden. This study was conducted in 6 of these centres.

### Procedure

A semi-structured interview guide consisting of open-ended questions was developed by the research group. It was tested in one interview resulting in some minor changes. The questions addressed the risks of ETS exposure, CHC nurse’s support to parents concerning quitting smoking or protecting their child from ETS exposure, the experience of answering the SiCET and the experience of obtaining a urine sample from the child, if the intervention had influenced them, and their thoughts about smoking in the future. There were also attendant questions like “tell me more about …” or “what did you think …” aiming to encourage the responders to explain their answers.

Recruitment and consent took place as follows. The nurses who had participated in the intervention were asked to approach parents who had fulfilled the whole year in the intervention and ask if they were willing to be interviewed about their experiences.

The CHC nurses obtained both verbal and written information about the purpose of the study and distributed the information to the parents who had participated in the intervention. The parents were asked if they wished to be contacted about taking part in this new study. Two of the authors then contacted the parents and asked if they agreed to be interviewed. The parents decided the time and place for the interviews. The interviews took place within 2 months after the parents had finished their participation and were performed either at a CHC centre (n = 8) or in the parents’ home (n = 3). The interviews were held in May to September 2011. They were recorded and transcribed verbatim.

### Analysis

The material was analysed by two of the authors, and based on an inductive conventional content analysis according to Hsieh and Shannon [16]. The interviews were read several times to obtain a general impression of the text. Codes were derived by words from the text that captured key thoughts or concepts. Notes were made about impressions and analysis. Codes were labeled and emerging subcategories were sorted into categories (Table 1). These stages were undertaken independently by two of the authors. Their analyses were then compared and discussed until consensus was reached. Afterwards, the analysis was discussed and confirmed by all authors in order to validate the findings. Finally, the interviews were read again in order to interpret the interviews as a whole.

**Table 1:** An example of the analysis process

**Code**	**Label for code**	**Subcategory**	**Category**
“It`s an eye-opener, it does become more real when you have to fill it in.. and you think of the number of cigarettes you smoke everyday and when do I smoke and things you wouldn`t reflect upon otherwise. You just do it out of habit”	Eye-opener	Immediate effect	Influence on smoking habits

### Ethical considerations

The intervention study, including evaluation, was approved by the research ethics committee in Linköping, Sweden (application registration number: Dnr M114-07). The study described in this article was performed as a master thesis by students in a specialist nursing program. According to Swedish law [17], ethical approval is not required for research studies conducted during advanced educational programs, but all considerations were given to ethical laws and guidelines.

Permission to perform the study was given by the heads of the participating CHC clinics.. The principles of the Declaration of Helsinki were followed throughout the study [18]. The participants could not be identified in any published material. It was made clear that the authors performing the interviews were not involved in the project and it was important that both good and bad experiences were expressed. The respondents got repeated verbal and written information about the study and gave their verbal consent to participate. The parents had earlier given their written consent to participate in the intervention study and a verbal consent to be interviewed about their experiences from this was regarded as satisfying. They were informed that they could withdraw from the study whenever they wanted and that confidentiality was guaranteed. The interviewers focused on the respondents’ participation in the intervention and not on their smoking habits.

## Results

Fifteen parents agreed to take part in the study. Four of them later withdrew, resulting in a study sample comprising eleven parents, two men and nine women. The respondents were 23– 57 years old, median 34 years. Education varied from high (academics, n = 3), middle (12 years at school, n = 5), to low (compulsory school, n = 3). All but one were cohabiting, and nine of them were working or studying. One of the participants had a non-Swedish background, but no interpreter was needed.

The findings revealed four categories: Receiving support, Respectful treatment, Influence on smoking habits, and Receiving information (Table 2).

**Table 2:** Subcategories and categories

**Subcategories**	**Categories**
- Understanding	Receiving support
- Offering help	
- Accepting a ‘no’	Respectful treatment
- No pressure	
- Immediate effect	Influence on smoking habits
- Moving in the process of change	
- CHC`s authority	
- A task for CHC	Receiving information
- Information to all	
- Motivational factors	
- Lack of knowledge	

### Receiving support

The first category describes the CHC nurses’ method for supporting the parents participating in the intervention. Two subcategories, *understanding* and *offering help*, were identified.

#### *Understanding*

The respondents talked about their CHC nurses as being understanding, interested and always doing their best for their child. They were found to be kind, empathic, and easy to talk with. Some of the parents expressed their own feelings of guilt for being a smoker and they were happy that the meeting with the CHC nurses did not increase their guilt.

They felt that the CHC nurses cared and wanted to help them.. The respondents received much encouragement from their CHC nurse if they stopped smoking or changed their smoking habits. The CHC nurse continued to ask about smoking even after the parents had stopped.

*“but I feel that I have had a lot of support. The CHC nurse was easy to talk to, and listened and was willing to help. I think that was good”* (Respondent no. 11)

#### *Offering help*

The respondents had positive experiences of the way they had been offered help. They received different types of advice about nicotine replacement, how to stop smoking, and where to find information on the Internet. If the CHC nurse could not offer the help the parents asked for, she helped them find it elsewhere, for instance by referring them to a specialist in smoking cessation.

*“I’ve had a great deal of information about possible options and…if the nurse at the child health clinic didn’t know the answer, she looked it up…or forwarded the query”* (Respondent no. 6)

Several parents said that they were offered support in different ways, although they did not want it as they could handle it on their own and they were not comfortable with receiving help.

*“Yes well, had I wanted more support, I would have received that. But I have more or less said that…..I want to….do it on my own.”* (Respondent no. 1)

### Respectful treatment

The second category describes the way the parents felt that they had been treated by the CHC nurse . Two subcategories were found; the CHC nurse showed them respect by *accepting a ‘no’*, and *no pressure*.

#### *Accepting a ‘no’*

The feeling of being able to say ‘no’ to further information or discussion about smoking was important for the parents. Quitting smoking was described as a long process that cannot start until one feels ready for it. It depended on where they were in the process of change. All the parents said that they could only do it when they felt ready.

*“We have also talked about how much I want to and how determined I am to quit and, as I said, it is not the right time yet. I’m thinking about it but I’m not ready to take action yet..”* (Respondent no.3)

The parents described the importance of CHC nurses showing respect for their autonomy in their decisions about smoking. If the parents cancelled meetings with a smoking cessation specialist, the CHC nurse accepted their decision.

#### *No pressure*

The respondents described a positive feeling of not being pushed by their CHC nurse. Some of the parents described previous bad experiences of health care personnel, who had nagged, condemned or criticised their smoking, or had urged them to quit smoking. This had given them a feeling of being a bad parent, which led to obstinacy, and smoking cessation became even more impossible.

*“No. Nagging doesn’t work on me, well…it has the opposite effect so to speak. It was sort of more ….they tried to force it on me.”* (Respondent no.7)

### Influence on smoking habits

The third category describes the extent to which the parents were or were not influenced by their participation in the intervention study. The subcategories found were *immediate effect*, moving in the process of change and effect due to *the CHC’s authority.*

#### *Immediate effect*

The parents described how they were influenced by answering the SiCET. It worked as an eye-opener, giving them second thoughts about their smoking habits.

*“…It is an eye-opener, it does become more real when you have to fill it in…and you think of the number of cigarettes you smoke every day and when do I smoke and things you wouldn’t reflect upon otherwise. You just do it out of habit.”* (Respondent no.6)

The respondents found the SiCET to be something that CHC could use in dialogues with all parents about smoking, since people are not always completely honest about their smoking. Some of the respondents had discussed their answers to the SiCET with the CHC nurse.

Parents described how they easily denied that their child was exposed to nicotine, but the result of the urine-cotinine test was seen as irrefutable evidence that such exposure had indeed occurred.

*“Deep down you still want to think that it is no problem, but if you then have it in black and white, you become more influenced on a personal level to quit.”* (Respondent no.1)

Respondents suggested that all parents should be offered a test on their children’s urine cotinine level, whether the parents were smokers or not, since relatives or friends in the child’s environment might be smokers.

#### *Moving in the process of change*

The parents described how they were influenced in different ways in the process of change Steps in the changing process were described as either the start of thinking about a change or taking the last step to smoking cessation. Some said they had changed their smoking habits and/or reduced the number of cigarettes per day due to participation in the study.

*“We don’t smoke as much now as we used to….so we have made that change and I suppose we’ll remain at that stage for a while before we can make the next change.”* (Respondent no.10)

Some of the parents said that they were not influenced by the study as they were already doing what they could to protect their child from ETS exposure. They were smoking outdoors with the door closed and they were not ready to take the next step in the process of change. Some said that family reasons, consequence to their health and costs had influenced them more than CHC.

#### *CHC authority*

The parents claimed that they really listened to the CHC nurses. They were trustworthy and parents had confidence in what they said.

*“A nurse at the child health care clinic - as a parent you listen to her, she knows everything. We ask her about all our problems, and I think a lot of parents do the same..”* (Respondent no.10)

### Receiving information

The fourth category describes the respondents’ thoughts about their knowledge of the consequences of ETS exposure and where to find reliable information. Four subcategories were found: *a task for CHC*, *information to all, motivational factors* and *lack of knowledge*.

#### *A task for CHC*

The respondents said that information about the consequences of ETS should be given by the CHC nurse since they are professionals and are supposed to have updated knowledge about smoking. They also have the child’s best interests and health in focus.

*“I have to say I think it’s quite natural that it goes that route (via the nurse) really, because I can’t see where else..”* (Respondent no.5)

Some parents said that they were used to hearing about the consequences of smoking when visiting health care clinics and that they did not really listen. However, when it came to consequences for their child they listened more carefully.

*“Us smokers are that stubborn we turn a deaf ear as soon as it is brought up, but you do stop and think when you hear (the child’s name), sure you do.”* (Respondent no.5)

#### *Information to all*

The respondents stressed that information should be given to all parents and not only to known smokers. Not all parents are honest and tell the CHC nurse that they smoke. There may be relatives and friends in the child’s environment that are smoking even if the parents do not. Some of the parents said that CHC has the potential to spread knowledge about smoking in the society.

*“They could have a family member who smokes and then they can inform that person about what happens to children if you smoke near them…”* (Respondent no.8)

#### *Motivational factors*

Parents said that they wanted to protect their child from ETS exposure. Increased knowledge about the consequences of ETS exposure on children can work as a motivational factor, increase willingness to stop smoking or encourage parents to make bigger efforts to protect children from ETS exposure.

*“Even if I’ve been a bad mum who smokes, I always think about protecting the children, I never smoke inside or in the car*.” (Respondent no.3)

*”But if you’ve had information on passive smoking and your children are the things dearest to you, then you find out what they have to put up with….you can take that on board if you want to stop smoking and that can make it easier …it can be a motivating factor.”* (Respondent no.7)

#### *Lack of knowledge*

The parents had problems answering the question on what they knew about health consequences for children due to ETS exposure. Few of them had been informed about these matters by the CHC nurse. Some of the parents had only received a brochure from the nurse and had not discussed the content. Some said they might have been offered information but had answered that they already knew all about it, while some thought they had not received any information. Most of them wanted more information about the consequences of ETS exposure on children. Parents who claimed that they were well informed about the consequences of ETS exposure had obtained the information from reading scientific articles on their own.

*“There isn’t that much information about it except that it is bad, so to speak. You haven’t been told why or how much goes into the mother’s milk.”* (Respondent no.6)

*”Perhaps she offered me a lot, I can’t swear she didn’t…but sometimes you really don’t want to listen, and then no one’s told you. That’s how it is.”* (Respondent no.2)

## Discussion

The findings indicate that the parents were satisfied with the meetings, including dialogues about smoking, with the CHC nurses as they treated them with support and respect. Their autonomy was respected in the dialogues and the CHC nurse understood where they were in the process of change. The findings also show how the respondents were influenced in a variety of ways by the intervention. Some of the parents had not obtained, or were not aware of having obtained, information about the consequences of ETS on children, and the parents’ knowledge of these consequences was sparse.

Some of the respondents expressed a feeling of stigmatization by society as being a bad parent because they smoked. This is in concordance with the findings of Irwin and colleagues [19], who found that the guilt and shame parents felt if others found out that they were smoking was worse than their child's exposure to ETS. Dietz et al. [20] found that cigarette smoking tends to be underreported by mothers because of the stigma associated with smoking. Irwin et al. [19] showed that the women openly shared their guilt and shame in the hope of receiving a sympathetic response and thereby shielding themselves from the judgment of others.

The findings show that the parents wanted more information about the health consequences for their children, as this could motivate them to give up smoking. This is in concordance with Lendahls et al. [21], who showed that all respondents were ready to receive information and wanted facts about negative health effects. Several of the parents interviewed claimed that they had received little or no information about this issue. There may be various explanations for this. As in Irwin et al. [19], the parents in our study might have given the impression of having good knowledge and the CHC nurse may not have wanted to destroy that image by checking what they actually knew. The parents in our study reported a high level of respect and empathy from the CHC nurse, and nurses might have been afraid of damaging their relationship with the parents, as described by Baxter et al. [22].

“Smoke-free children”, a national intervention in Sweden in the 1990s, stressed that the first step always was to ask what the parents knew about the effects of smoking on children [23]. Several studies have shown a positive relationship between low educational level and low awareness of ETS risks on children [24,25], and this might explain our findings since only three parents had an academic background.

The difference between nagging and being interested in the parents’ smoking habits can be subtle and may be perceived diversely by different parents. Our findings indicate that the parents found that most of the CHC nurses in our study struck a good balance between nagging and showing interest. The parents found the SiCET to be a good way for the CHC nurse to promote discussion of smoking habits.

Some of the parents in our study said they were influenced by the intervention and had changed their smoking habits, while others had started to think about a change. Some parents said they were not influenced at all; they already did what they could to protect their child or were not motivated to make a change. This can be explained by Prochaska and DiClimente [26], who stress that a person can be more or less adaptive and influenced to make a change depending on where they are in the process of change. Their theory also emphasises that there are several steps in the process of change prior to a real behaviour change and that it is important to find the best course of action when meeting persons on different steps.

### Study strengths and limitations

A strength of this study was that the authors who conducted the interviews and analysed the results were not involved in the intervention and were unknown to both nurses and parents. The authors performed the analysis by themselves and then compared their findings and found concordance, which strengthens credibility*.* A further strength was that the respondents included men and women and they represented different ages, educational levels and professions. People who had stopped smoking or changed their smoking habits, and people who had not made any changes were also represented. All this strengthens the transferability and trustworthiness of the study.

A limitation of the study was that it was not possible to include non-Swedish speaking parents in the study, since we had no interpreter for the interviews. This is a well known challenge in qualitative studies where every shade in the language is important, and therefore the ability to use your mother-tongue is crucial.

## Conclusion

The findings indicate that the parents participating in the intervention were satisfied with most of it. The way the CHC nurses treated them with respect and supported them in different ways to stop smoking or change their smoking habits was positive. The SiCET was seen as an eye-opener about their smoking habits, and the urine test was considered to give irrefutable evidence that their child had been exposed to ETS. Few of the parents claimed that they had been informed by the CHC nurse about the health consequences of ETS exposure.

The clinical implication is that CHC is an important arena for preventive work aiming to minimize children’s exposure to tobacco smoke. CHC nurses can play an important role in tobacco prevention, but should be more explicit in their communication with parents about tobacco issues. The SiCET was referred to as an eye-opener and can be useful in the MI dialogues performed by nurses in order to support parents in their efforts to protect their children from ETS.

## Competing interests

None of the authors have in the past five years received reimbursements, fees, funding, or salary from an organization that may in any way gain or lose financially from the publication of this manuscript, either now or in the future. No such organization have been financing this manuscript (including the article-processing charge).

None of the authors hold any stocks or shares in an organization that may in any way gain or lose financially from the publication of this manuscript, either now or in the future.

None of the authors hold or are currently applying for any patents relating to the content of the manuscript. None of the authors have received reimbursements, fees, funding, or salary from an organization that holds or has applied for patents relating to the content of the manuscript.

None of the authors have any other financial competing interests.

None of the authors have any non-financial competing interests (political, personal, religious, ideological, academic, intellectual, commercial or any other) to declare in relation to this manuscript.

No funding was received for this study.

## Authors’ contributions

HA, MR and AKJ participated in the design of the study and acquisition of data. All authors participated in analysis and interpretation of data, helped to draft the manuscript and read and approved the final manuscript.

## Pre-publication history

The pre-publication history for this paper can be accessed here:

http://www.biomedcentral.com/1471-2431/14/69/prepub
